# Building blocks for automated elucidation of metabolites: natural product-likeness for candidate ranking

**DOI:** 10.1186/1471-2105-15-234

**Published:** 2014-07-05

**Authors:** Kalai Vanii Jayaseelan, Christoph Steinbeck

**Affiliations:** 1 Cheminformatics and Metabolism, European Molecular Biology Laboratory - European Bioinformatics Institute (EMBL-EBI), Wellcome Trust Genome Campus, Hinxton, Cambridge CB10 1SD, UK

**Keywords:** Computer-assisted structure elucidation, Metabolomics, Natural product-likeness

## Abstract

**Background:**

In metabolomics experiments, spectral fingerprints of metabolites with no known structural identity are detected routinely. Computer-assisted structure elucidation (CASE) has been used to determine the structural identities of unknown compounds. It is generally accepted that a single 1D NMR spectrum or mass spectrum is usually not sufficient to establish the identity of a hitherto unknown compound. When a suite of spectra from 1D and 2D NMR experiments supplemented with a molecular formula are available, the successful elucidation of the chemical structure for candidates with up to 30 heavy atoms has been reported previously by one of the authors. In high-throughput metabolomics, usually 1D NMR or mass spectrometry experiments alone are conducted for rapid analysis of samples. This method subsequently requires that the spectral patterns are analyzed automatically to quickly identify known and unknown structures. In this study, we investigated whether additional existing knowledge, such as the fact that the unknown compound is a natural product, can be used to improve the ranking of the correct structure in the result list after the structure elucidation process.

**Results:**

To identify unknowns using as little spectroscopic information as possible, we implemented an evolutionary algorithm-based CASE mechanism to elucidate candidates in a fully automated fashion, with input of the molecular formula and ^13^*C* NMR spectrum of the isolated compound. We also tested how filters like natural product-likeness, a measure that calculates the similarity of the compounds to known natural product space, might enhance the performance and quality of the structure elucidation. The evolutionary algorithm is implemented within the SENECA package for CASE reported previously, and is available for free download under artistic license at http://sourceforge.net/projects/seneca/. The natural product-likeness calculator is incorporated as a plugin within SENECA and is available as a GUI client and command-line executable. Significant improvements in candidate ranking were demonstrated for 41 small test molecules when the CASE system was supplemented by a natural product-likeness filter.

**Conclusions:**

In spectroscopically underdetermined structure elucidation problems, natural product-likeness can contribute to a better ranking of the correct structure in the results list.

## Background

A significant number of the small molecules involved in the metabolism of organisms is still unknown. Methods for the structure elucidation of unknown metabolites are therefore needed. Computer-assisted structure elucidation (CASE), a field that has been researched for over 40 years [1], lends itself to this end. For complex structure elucidation problems, a rich set of mass spectral as well as 1D and 2D NMR spectroscopic information is required [2].

Under high-throughput conditions, however, only spectroscopic information that can be recorded quickly is used. Therefore, successful structure determination based on a limited set of spectroscopic information is important. 1D proton NMR is the most commonly used NMR method in metabolomics; however, in the present study, we based the structure elucidation on carbon-13 NMR because reliable open-source methods for proton prediction, including the coupling constants required to produce realistic 1D spectra, are not available. It is common knowledge that 1D NMR alone is usually not sufficient to establish the identity of a hitherto unknown compound.

Recently, we and others demonstrated that metabolites and natural products cluster in a distinct area in structure space [3,4], leading to the concept of a calculated natural product (NP)-likeness.

Here, we investigated whether NP-likeness can be used as another component to the fitness evaluators to help elucidate candidates with minimum spectral information and increase the likelihood of a natural product ranking highly in a set of solutions. The methods presented here have been implemented in the SENECA system, an open-source java-based desktop application to perform CASE for organic molecules. The application takes a molecular formula generated from high-resolution mass spectrometry and spectral data from a suite of NMR experiments and performs a stochastic search in the constitutional space, guided by a fitness function. With the above input, the ability of the application to produce the correct structure for candidates with up to 30 heavy atoms has been demonstrated previously [5]. Other systems have been shown to solve even larger problems, but these systems are closed-source and proprietary [6].

## Methods

SENECA has been refactored completely and is now based on the Chemistry Development Kit (CDK), a popular open-source library for cheminformatics [7,8]. The CDK contains a graph-based, object-oriented representation of molecules and provides flexible functionalities to perform various operation on molecules, including valence electrons check, ring perception, aromaticity detection, mutation, and atom environment representation. SENECA performs a stochastic search of constitution space (the space made up of all chemical compounds with the same gross formula), guided by a fitness or scoring function, which contains terms that evaluate the similarity of predicted and experimental spectra and terms that evaluate the validity of the currently inspected solution. SENECA contains implementations of simulated annealing [5] and an evolutionary algorithm. The latter is a completely new implementation and not related to the one reported previously [9] that was based on an older, obsolete library and that therefore was discontinued.

### Availability

The latest version of SENECA is available both as a desktop application and as a command-line executable. SENECA is available for free download under artistic license from SourceForge [10].

### Evolutionary algorithm for stochastic search

**Figure 1:**
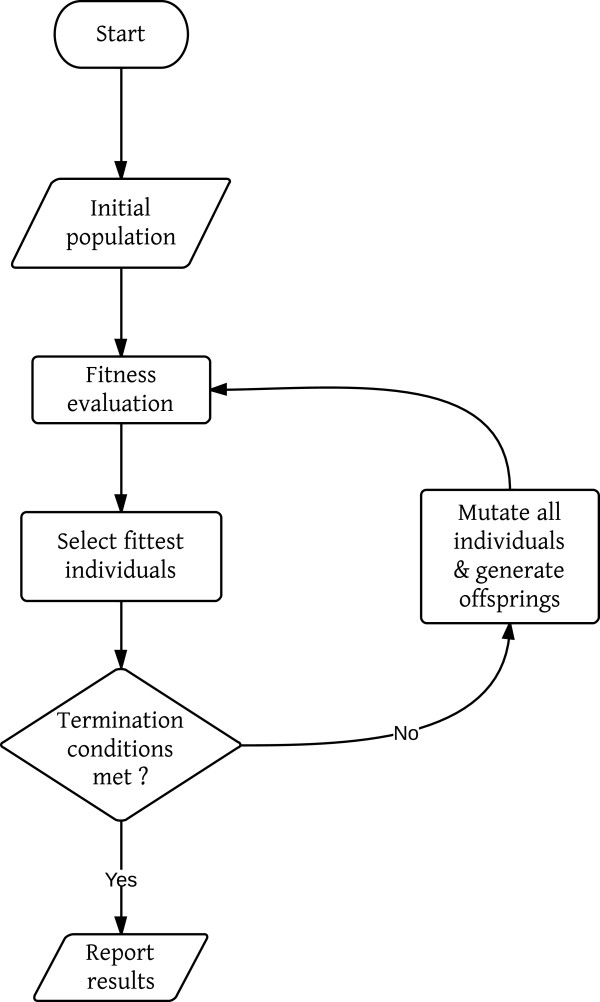
**Evolutionary algorithm scheme for CASE.** Evolution starts with an initial population of 16 individuals. The initial population is seeded by mutating the single random structure generated from the molecular formula. The population is evaluated for termination criteria, i.e. maximum fitness, maximum allowed runtime, maximum allowed generations, or maximum allowed generations with no improvement. Evolution continues until any one of the above conditions is met. The population is doubled by mutating every individual before fitness is evaluated. After fitness evaluation, the fittest ones are promoted to the next generation by round-robin tournaments selection. Once one of the termination conditions is met, the solution set is reported.

Evolutionary algorithms (EA) are based on the Darwinian principles of evolution, incorporating the concepts of natural selection and survival of the fittest. The overall scheme implemented by us for the optimization problem is similar to the scheme reported in [9], which has both mutation and recombination operators. In our scheme, we used only the mutation operator because it offered the desired results and was easier to configure. The simple EA scheme implemented by us is illustrated in Figure 1.

In our EA scheme, evolution starts with a population of a configurable number of randomly generated individuals, seeded by an initial molecular structure (connected graph) derived from the input molecular formula. To generate an initial population, the initial seed is mutated randomly by the mutation functionality within the CDK based on the *Faulon* mutation operator [11]. Each individual is evaluated for fitness by our fitness evaluators and cross checked with termination conditions. The program can be configured to terminate if any of the following criteria are reached: maximum fitness, maximum allowed runtime, maximum allowed generations, or maximum allowed generations with no improvement. Here, we used only one of the termination conditions, namely the maximum allowed generations, because then we could collect all the isomers traversed and rank them to see if there was significant improvement in the ranking of the exact structure with the application of an NP-likeness filter. If the termination conditions are not met, each individual is mutated to generate one offspring, thereby doubling the population. The doubled population is evaluated again for fitness and the fittest individuals are chosen by round-robin tournament selection. If there is no improvement in fitness for five consecutive generations, fresh individuals are added to the population using a vicinity search around the top two fittest individuals. After the niche search the population is expanded from 16 to 24 and allowed to run for five generations. If again no fitness improvement is recorded, the population size is reduced back to 16 using tournament selection. The above evolution procedure is repeated until the termination conditions are met (default maximum allowed generations was set to 3000). Once met, the evolution is stopped and the elucidated candidates are reported.

### Fitness evaluators

A suite of fitness evaluators (judges), each of which evaluates a certain optimization criterion, e.g. agreement between the recorded chemical shift value from various NMR techniques (^13^*C* NMR, HHCOSY, HMBC, and HSQC) and the observed structural features in the candidate, has already been presented in SENECA [5,9]. The scoring methods used by these judges are not described in detail here; however, the newly implemented fitness evaluators that have not been reported previously are discussed below.

#### NMRShiftDB judge

NMRShiftDB is an open-access, open submission database that houses about 42,000 organic molecules and their associated recorded experimental 1D NMR spectra [12]. The database can be accessed at http://nmrshiftdb.org.

The HOSE (Hierarchical Organisation of Spherical Environments) code [13] is a canonical way of capturing atom environments in a linear extendable fashion. A fitness evaluator from a previous version of SENECA [5] used the 651 one-sphere HOSE - ^13^*C* shift entries published by *Bremser*[14] to keep the hybridization and heteroatom attachments of the predicted structures in check. The prediction precision can be improved if the extended environment of atoms can be captured by increasing the HOSE sphere height [5]. The NMRShiftDB database is used to produce an index of HOSE with extended heights (from 1 to 4) and to associate it with a mean chemical shift and a confidence score, similar to *Bremser’s* approach [14].

The mean shift value for each HOSE code is calculated by averaging all the observed shifts over the molecule occurrence count. The standard deviation, therefore, will increase as the sphere height decreases. The confidence limit for each mean shift is calculated by multiplying the standard error with the two-sided Student’s t-distribution value for 95% confidence. If the confidence limit is less than 5 ppm, a minimum value of 5 ppm is assigned. The index generated serves as a lookup table for the new NMRShiftDBJudge.

The NMRShiftDB judge calculates the ^13^*C* spectrum for the candidate and assigns a fitness score based on its agreement with the experimental spectrum as shown in *equation*1. If the back-calculated (*δ*_*p* *r* *e* *d*_) and the absolute deviation of a given experimental shift (*δ*_*e* *x* *p*_) is within the confidence limit (CL) offered for that shift value, full points (*P*_*m* *a* *x*_) are awarded for that carbon atom, otherwise zero. The summation of all the carbon atom scores gives the total score, validating the agreement between the ^13^*C* spectrum and the expected atom environments. *Equation*2 can be applied to normalize the score between 0 and 1.

(1) $$\;{\text{Score}}_{\text{nmr}\_\;\text{judge}}=\underset{\text{Carbons}}{\sum }\left\{\begin{array}{cc} P_{\text{max}} & \;\text{if}\;\left(\delta_{\text{pred}}-\delta_{\text{exp}}\right)\leq \text{CL}\\ 0 & \;\text{if}\;\left(\delta_{\text{pred}}-\delta_{\text{exp}}\right)>\text{CL} \end{array}\right)$$

(2) $${\text{NMRcost}}_{\text{norm}}=\frac{{\text{Score}}_{\text{nmr}\_\;\text{judge}}}{\text{Carbon}\_\text{count}*P_{\text{max}}}$$

#### NPLikeness judge

With only NMRShiftDBJudge many isomers will emerge as the fittest by satisfying all the fragment-to-shift associations. This is a classic problem in CASE, i.e. the possibility of finding not one but many structural candidates that satisfy the given NMR constraints. Then expert opinion is relied on to pick the correct candidate solution. Hence, candidate ranking methods are indispensable in CASE when numerous solutions are given.

An automated way to rank these candidate solutions is to check their structural validity, for example by calculating their structural similarity with known NPs. Therefore, inclusion of a NPLikenessJudge is expected to improve the ranking of the candidates by penalizing improbable structures on the fly during evolution. To make decisions on the NP-likeness of a molecule, knowledge about the structural space of known NPs as opposed to synthetics is used. Previously, we reported such a scorer based on the open-data of known natural products and synthetics [4], with the main motivation of including it in the CASE program. We implemented a NPLikenessJudge by including the NP-likeness calculator within SENECA. The judge scores molecules for NP-likeness based on the occurrence of its fragments in the known natural product fragment space. The NP score given is between -3.5 and +3.5, where a higher score indicates greater NP-likeness. This score is normalized between 0 and 1 using *equation*3, where *max* and *min* are the most extreme values given by the NP-likeness scorer.

When NPLikenessJudge is used along with NMRShiftDBJudge the total cost is calculated using *equation*4. Thus, the candidate molecule is weighted more for NMR agreement and less for NP-likeness.

(3) $${\text{NPcost}}_{\text{norm}}=\frac{{\text{Score}}_{\text{np}\_\;\text{judge}}-\text{min}}{\text{max}-\text{min}}$$

(4) $${\text{Cost}}_{\text{total}}=\left[\frac{{\text{NMRcost}}_{\text{norm}}\left(1+{\text{NPcost}}_{\text{norm}}\right)}{2}\right]$$

#### AntiBredt Judge

Another way to filter improbable structures during evolution is to check for structures that are sterically constrained. AntiBredtJudge penalizes a structure if it has a double bond at bridgehead atoms in rings with seven or less atoms. An algorithm to detect these unrealistic structures was reported previously [15] and this algorithm has been implemented in our CASE scheme.

### Test case collection

**Figure 2:**
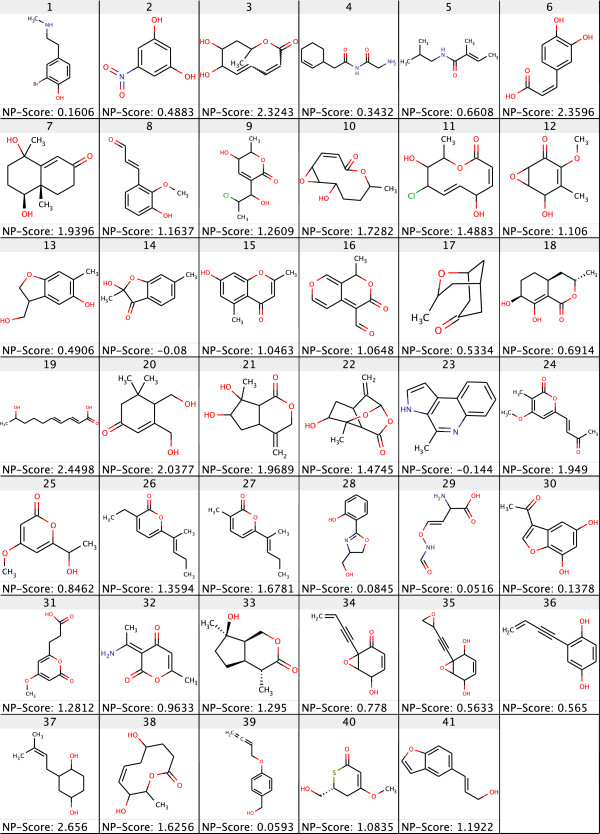
**Forty-one test structures collected from the *Journal of Natural Products*.** Test molecules with a heavy atom count of ≤15 were collected only from recently published articles. All these structures were cross checked with the NMRShiftDB index to ensure that none of them was already present in the training index. The NP-Score for the given molecules were calculated using our previously reported NP-likeness calculator and is based on 3-sphere signature height.

To test the performance of the CASE system in predicting the correct structures using only the ^13^*C* spectrum, we collected 41 test cases from the *Journal of Natural Products*. We selected molecules with heavy atom counts ≤ 15 from recently published articles. From each article, the molecular formulae (MF), ^13^*C* chemical shifts, and DEPT 90 and DEPT 135 assignments were extracted to determine the number of hydrogen atoms attached to carbon atoms and then the published molecular structure was extracted for final cross validation. The MF and spectrum information were entered manually into the SENECA GUI client and saved as a SENECA markup language (SML) file, SENECA’s native file format based on XML. The molecular structure information was drawn using a structure drawer and saved as an MDL mol file to be used for identifying the correct structure in the solution set at the end of the CASE. All the structures were cross checked with the NMRShiftDB index to ensure that none of them were already present in the index. The collected test cases along with their NP-likeness score are illustrated in Figure 2.

## Results and discussion

### Performance of the CASE system

The performance of SENECA was tested using only the new fitness evaluators NMRShiftDBJudge, NPLikenessJudge, and AntiBredtJudge. To test the enhancement in candidate ranking with the application of NP-likeness, we performed two different evaluations for the 41 test cases. In the first evaluation (*NMR_only*), only two of the judges, NMRShiftDBJudge and AntiBredtJudge, were used, and in the second evaluation (*NMR_NP*) all three judges were used. The stochastic search does not guarantee that the global optimum will be found in one run; therefore, it is common to collect the results from several runs, and then combine and rank them. To test the performance of the CASE system, we performed 100 sequential runs for every test case, i.e. 4100 runs for each of the *NMR_only* and *NMR_NP* evaluations. Each run performs an evolution that terminates after 3000 generations and reports back all the elucidated candidates.

**Figure 3:**
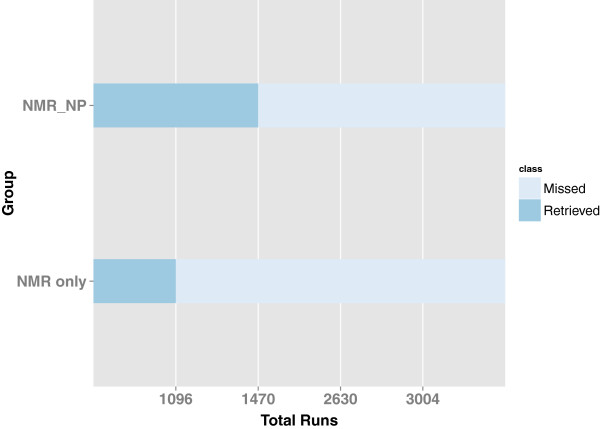
**Performance of SENECA using the new fitness evaluators.** 100 sequential runs were performed for every 41 test case, i.e. 4100 runs each for the *NMR_only* and *NMR_NP* evaluations. The *NMR_NP* judge predicted the correct solutions more often than the *NMR_only* judge. Correct candidates were retrieved in the solution set in 1470/4100 runs, and 1096/4100 runs, using *NMR_NP* and *NMR_only*, respectively.

**Figure 4:**
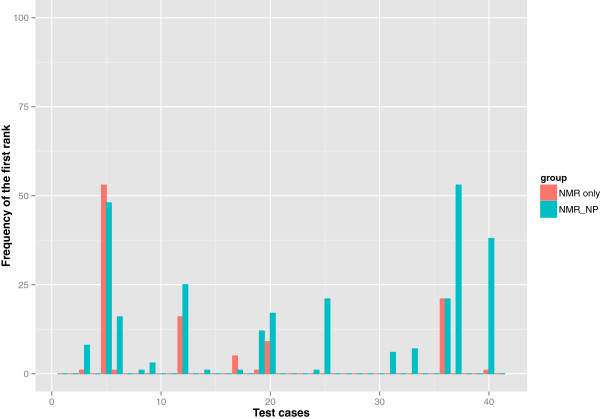
**Number of times the correct candidate was ranked first among the retrieved cases.** Correct structures were predicted for 36/41 test cases in total. Of the correctly predicted cases, 9/34 and 17/35 were ranked first using the *NMR_only* and *NMR_NP* judges, respectively, showing that the application of NP-likeness frequently improved the rank of the correct solution.

**Figure 5:**
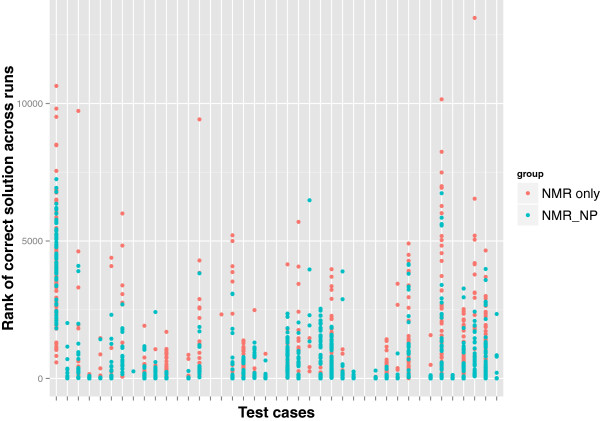
**Spread of ranks of all the correct solutions across all test cases and runs.** The rank given for the correct structure among the other predicted candidates using the *NMR_only* and *NMR_NP* judges is shown. An empty index indicates that there was no successful prediction for that test case in any of the 100 runs.

**Figure 6:**
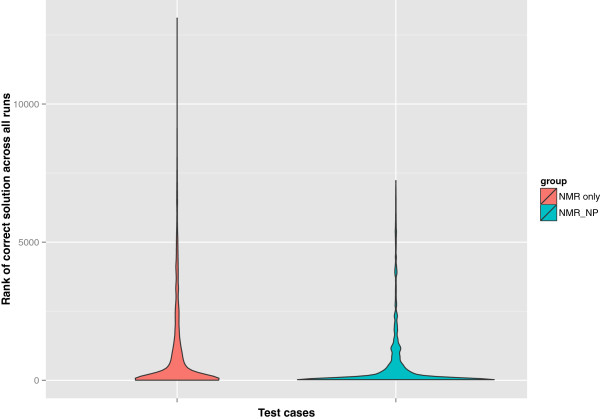
**Distribution of ranks of all the correct solutions across all test cases and runs.** The overall distribution of ranks given to the correct structure as shown in Figure 5, is summarised here using a violin plot.

**Figure 7:**
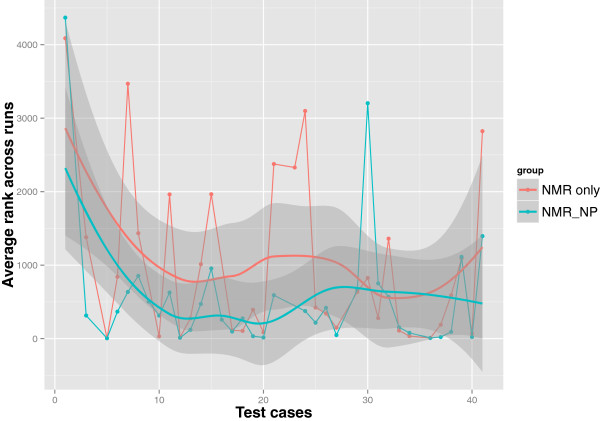
**Average rank of all the correct solutions over the 100 runs for each test case.** In addition to predicting the correct structure more often (as shown in Figure 3), the application of NP-likeness, on average, improved the rank of the correct structure among the predicted candidates. The average ranks here are smoothened out by applying loess regression.

We observed that the frequency of elucidating the correct structure in the solution set was higher with the three *NMR_NP* judges than with the two *NMR_only* judges as shown in Figure 3. Although the retrieval percentage was low because of the spectroscopically underdetermined problems among the test cases, there was a 9.1% overall improvement in the predictions of correct candidates when NPLikenessJudge was included as a judge in the evaluations. Correct structures were retrieved in the solution set for 36 of the 41 cases with the application of all our fitness evaluators. The five cases for which no correct solution was found are numbered 2, 4, 22, 28, and 35 in Figure 2. The most interesting question is: in how many of the 36 retrieved cases was the correct candidate ranked the highest and how frequently did this happen? As illustrated in Figure 4, nine out of 34 cases and 17 out of 35 cases were ranked the highest using the *NMR_only* and *NMR_NP* judges, respectively, indicating that the application of NP-likeness frequently improved the rank of the correct solution. The improvement in ranking through the application of NPLikenessJudge is also indicated in Figure 5 and Figure 6, where the overall rank distribution is plotted for all test cases across all runs where there was a correct structure prediction. The empty indices in Figure 5 indicate that there was no correct prediction for these test cases in any of the 100 runs. Figure 6 using a violin plot summarises the distribution of overall ranks shown in Figure 5. The averaged-out rank distribution in Figure 7 shows how application of an NP-likeness filter tended to improve overall the rank of the correct solution among other predicted candidates.

## Conclusion

Here, the application of natural product-likeness to achieve a higher ranking of the correct compound in automated structure elucidation of natural products with an otherwise limited set of spectroscopic information is presented. The newly developed methods described in this article are implemented in the open-source SENECA package for CASE, which features an evolutionary algorithm for structure elucidation and is available as a GUI client or as a stand-alone command-line executable. Fitness evaluators based on ^13^*C* NMR spectrum-to-structure associations in the NMRShiftDB database [12] and an NP-likeness score [4] have been integrated in the scoring function of the evolutionary algorithm scheme. With our publicly available open-data based fitness evaluators we have demonstrated the successful elucidation of correct structures for unknowns in 36 of 41 test cases, and achieved the highest rank for the correct structures of 17 of the 36 cases. We have also shown that significant improvement in overall prediction frequency and average ranking can be achieved with the application of the NP-likeness filter. We believe that these results can lead to improvements in CASE systems for use in metabolomics data analysis pipelines. The open-source, open-data implementation allows other researchers to contribute to or modify the package, and use their own training data for fitness evaluation.

## Competing interests

The authors declare that they have no competing interests.

## Authors’ contributions

CS conceived the project and guided the development. KVJ conducted the study, selected the data, implemented the evolutionary algorithm scheme and new fitness evaluators within SENECA, and tested it. Both authors contributed to the manuscript draft, and read and approved the final manuscript.

## References

[B1] Steinbeck C **The automation of natural product structure elucidation** Curr Opinion Drug Discov Dev 2001 4 3 338 342 11560068

[B2] Steinbeck C Gasteiger J, Engel T **Computer-assisted structure elucidation** Handbook of Chemoinformatics 2003 Weinheim: John Wiley & Sons 1378 1406

[B3] Peironcely JE Reijmers T Coulier L Bender A Hankemeier T **Understanding and classifying metabolite space and metabolite-likeness** PloS one 2011 6 12 e28966 10.1371/journal.pone.0028966 22194963 PMC3237584

[B4] Jayaseelan K Moreno P Truszkowski A Ertl P Steinbeck C **Natural product-likeness score revisited: an open-source, open-data implementation** BMC Bioinformatics 2012 13 106 10.1186/1471-2105-13-106 22607271 PMC3436723

[B5] Steinbeck C **SENECA: A platform-independent, distributed, and parallel system for computer-assisted structure elucidation in organic chemistry** J Chem Inf Comput Sci 2001 41 6 1500 1507 10.1021/ci000407n 11749575

[B6] Blinov KA Carlson D Elyashberg ME Martin GE Martirosian ER Molodtsov S Williams AJ **Computer-assisted structure elucidation of natural products with limited 2D NMR data: application of the StrucEluc system** Magn Reson Chem: MRC 2003 41 5 359 372 10.1002/mrc.1187

[B7] Steinbeck C Han YQ Kuhn S Horlacher O Luttmann E Willighagen E **The Chemistry Development Kit (CDK): an open-source Java library for chemo- and bioinformatics** J Chem Inf Comput Sci 2003 43 2 493 500 10.1021/ci025584y 12653513 PMC4901983

[B8] Steinbeck C Hoppe C Kuhn S Guha R Willighagen EL **Recent developments of the Chemistry Development Kit (CDK) - an open-source Java library for chemo- and bioinformatics** Curr Pharm Des 2006 12 17 2111 2120 10.2174/138161206777585274 16796559

[B9] Han Y Steinbeck C **Evolutionary-algorithm-based strategy for computer-assisted structure elucidation** J Chem Inf Model 2004 44 2 489 498 10.1021/ci034132y 15032528

[B10] Jayaseelan KV Steinbeck C **SENECA - Sourceforge availability** [http://sourceforge.net/projects/seneca/]

[B11] Faulon JL **Stochastic generator of chemical structure. 2. Using simulated annealing to search the space of constitutional isomers** J Chem Inf Comput Sci 1997 36 731 740

[B12] Steinbeck C Kuhn S **NMRShiftDB - compound identification and structure elucidation support through a free community-built web database** Phytochemistry 2004 65 19 2711 2717 10.1016/j.phytochem.2004.08.027 15464159

[B13] Bremser W **HOSE - novel substructure code** Anal Chem Acta 1977 103 355 365

[B14] Bremser W **Expectation ranges of 13 C NMR chemical shifts** Magn Reson Chem 1985 23 271 275 10.1002/mrc.1260230413

[B15] Nuzillard JM **Quick method for Anti-Bredt structure detection** J Chem Inf Comput Sci 1994 34 723 724 10.1021/ci00020a004

